# Immunologic features of asymptomatic postvaccination infections with the Delta variant of SARS‐CoV‐2 in adults

**DOI:** 10.1002/iid3.670

**Published:** 2022-06-21

**Authors:** Rui Duan, Qiang Mao, Xu Ding, Qiwu Qiu, Pei Wang

**Affiliations:** ^1^ Department of Laboratory Medicine and Blood Transfusion The First People's Hospital of Jingmen Hubei China; ^2^ Department of Medical Records and Statistics The First People's Hospital of Jingmen Hubei China; ^3^ Department of Infectious Diseases The First People's Hospital of Jingmen Hubei China

**Keywords:** asymptomatic, breakthrough infection, Delta variant, immunologic features, postvaccination infections, SARS‐CoV‐2

## Abstract

**Background:**

Asymptomatic infections may play an important role in severe acute respiratory syndrome coronavirus‐2 (SARS‐CoV‐2) Delta variant transmissions. However, the immunologic features of asymptomatic postvaccination infections with the Delta variant of SARS‐CoV‐2 in adults remain to be defined.

**Methods:**

A retrospective study involving 36 vaccinated adults infected with the SARS‐CoV‐2 Delta variant was performed. Their demographic and laboratory data were collected and analyzed in The First People's Hospital of Jingmen from August 4 to 20, 2021.

**Results:**

Of the 36 adults, 6 persons had an asymptomatic infection. The severity of the SARS‐CoV‐2 infections was highly correlated with the doses of vaccinations (*p* = 0.019). The symptomatic and asymptomatic infected SARS‐CoV‐2 adults showed normal levels of leukocytes and lymphocytes. The C‐reactive protein (CRP) and interleukin‐6 (IL‐6) levels were elevated in the symptomatic groups. The period between the last vaccination to the time of infection in the asymptomatic group was longer than that in the mild and moderate groups (73 vs. 61 vs. 50 days; *p* = 0.047). The percentage of suppressor T‐cells in the asymptomatic group was the highest (32.2 ± 4.0% vs. 22.0 ± 7.2% vs. 29.3 ± 8.0%; *p* = 0.004). The signal‐to‐cutoff ratio value of total antibody against SARS‐CoV‐2 in the asymptomatic group was lower than that in the other two groups (383 vs. 703 vs. 1792; *p* < 0.001) and much lower than that in the moderate group. The multivariate ordinal logistic analysis after adjusting for gender, vaccination date, and vaccination dose indicated that CRP at Days 4−7 and 8−14, IL‐6 on Days 4−7, and total antibody were risk factors for coronavirus disease 2019 severity.

**Conclusion*s*:**

Asymptomatic postvaccination infections with the Delta variant of SARS‐CoV‐2 in adults tend to infect persons vaccinated twice. The immunophenotype profile for asymptomatic postvaccination infections is less inflammatory and accompanied by relatively lower antibody titers.

## Graphical abstract

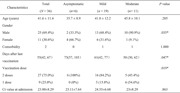

## 1 INTRODUCTION

Currently, the rollout of the Sinopharm (Vero cell) inactivated vaccine has been adopted for mass vaccination in China. It is reported that the protective efficiency of this inactivated vaccine against the severe acute respiratory syndrome coronavirus‐2 (SARS‐CoV‐2) Delta variant after two doses of vaccination is 59.0% and 70.2% against moderate SARS‐CoV‐2 infection, and 100% against severe SARS‐CoV‐2 infection.^1^ The Delta variant of SARS‐CoV‐2 was the dominant form of SARS‐CoV‐2 in the United Kingdom and many other countries during the summer and early autumn of 2021.^2^ Later on, the Delta variant was succeeded by the Omicron variant.^3^

There is evidence that many infections of SARS‐CoV‐2 are asymptomatic and that the virus can be transmitted during such infections.^4^ However, the impact of vaccination with inactivated virus vaccine on asymptomatic SARS‐CoV‐2 infections and the transmission risks remain unclarified.^5^ There are less data on postvaccination breakthrough infections with the Delta variant of SARS‐CoV‐2.^6^ The immunologic features of persons with asymptomatic postvaccination infections with the Delta variant of SARS‐CoV‐2 were studied and compared with the groups that had mild and moderate diseases.

## 2 MATERIALS AND METHODS

### 2.1 Study design and participants

To control the small outbreak of the Delta variant infections in Jingmen, people who had been in contact with real‐time reverse transcriptional polymerase chain (RT‐PCR) confirmed new cases of SARS‐CoV‐2 were placed in quarantine. The persons placed in quarantine were tested regularly and when tested positive they were transmitted to The First People's Hospital of Jingmen.

We enrolled a total of 36 patients from August 4 to 20, 2021 at The First People's Hospital of Jingmen. All patients were confirmed to have a SARS‐CoV‐2 infection by positive RT‐PCR on nasopharyngeal swabs. The Delta variant was identified as B.1.617.2 by sequence analysis. All participants were vaccinated with Sinopharm (Vero cell) inactivated coronavirus disease 2019 (COVID‐19) vaccine. The 36 participants were divided according to their presentation during hospitalization into three groups: asymptomatic group (*n* = 6), mild group (*n* = 19), and moderate group (*n* = 11). Antiviral treatment of 200 mg Arbidol, three times a day, was used for all SARS‐CoV‐2 study participants.

### 2.2 Definition of the severity of COVID‐19

The definition of disease severity of SARS‐CoV‐2 infections was based on the seventh version of the Chinese guideline for the management of COVID‐19.^7^ The asymptomatic patients, were defined as patients with a positive RT‐PCR and without clinical symptoms and signs, and without abnormalities of lung computed tomography (CT) at admission. These asymptomatic patients developed no lung CT abnormalities and had no complaints during a period of 14 days of hospitalization.^8^ Patients with slight clinical symptoms and without imaging findings of pneumonia were considered to have the mild condition. When patients had a fever or respiratory symptoms and imaging findings of pneumonia, they were identified as having a moderate condition.

### 2.3 RT‐PCR, total antibody against SARS‐CoV‐2 measurement, and other laboratory tests

Viral RNA was extracted from nasopharyngeal swabs with a nucleic acid kit (Roche) on an automatic workstation MagNA Pure 96 system (Roche). RT‐PCR with Applied Biosystems ViiA7 Dx (Applied Biosystems) and RT‐PCR reagent BioGerm (BioGerm) were commercially obtained and used for virus detection. The cycle threshold value (*C*_t_) obtained in the amplification of the N gene was recorded at admission. The total antibody (IgA, IgM, IgG) against SARS‐CoV‐2 in serum samples was determined by chemiluminescence microparticle immunoassay kits (Wantai). Leukocytes and lymphocytes cell counts were assessed at Sysmex XN‐9000 (Sysmex), and C‐reactive protein (CRP) was performed at Cobas C702 (Roche), interleukin‐6 (IL‐6) was tested at Cobas e602 system (Roche). Helper T‐cells (Th) (CD3+ and CD4+) and suppressor T‐cells (Ts) (CD3+ and CD8^+^) were assessed using the BD FACS Calibur^TM^ Flow cytometer (Becton Dickinson).

### 2.4 Data collection

Data including demographic data, vaccination history, medical history, symptoms, signs, laboratory tests, lung CT, and other information were collected and analyzed. The data were reviewed by a trained team consisting of physicians and technicians in The First People's Hospital of Jingmen.

### 2.5 Statistical analysis

A database was established and statistical analysis was performed with SPSS 22.0. The categorical variables were described as frequency rates and percentages, and continuous variables were described as means and SDs, medians, or interquartile ranges. The Kruskal−Wallis *H* test or *F* test was used to compare the continuous variables among three groups. Fisher's exact test was used to compare categorical variables. Univariable ordinal logistic regression was used to identify the association between leukocytes, lymphocytes, CRP, IL‐6, T‐cell subsets, and total antibodies with disease severity in patients with COVID‐19. Additionally, multivariate ordinal logistic regressions were conducted to assess the independent association of immunologic markers with disease severity in patients with COVID‐19, adjusting for potential confounders, taking the moderate group as the reference group. Results from regression models were expressed as odds ratio (OR) with 95% confidence intervals (CI). *p* Values lower than 0.05 were considered statistically significant.

## 3 RESULTS

### 3.1 Demographics and baseline characteristics of 36 adults infected after vaccination

Demographics and baseline characteristics of 36 persons are shown in Table 1. Of the 36 infected adults, 6 (16.7%) persons were asymptomatic, 19 (52.8%) had mild and 11 (30.5%) had a moderate illness. The median age of the group was 42 years and 25 (69.4%) were male. The proportion of males in the asymptomatic group was significantly different from that of the moderate group (33.3% vs. 68.4% vs. 90.9%, *p* = 0.035). Concerning age, there was no significant difference between the three groups (*p* = 0.205). The vaccination degree was different among three groups. The asymptomatic group had received two doses of the vaccine and was fully vaccinated. Disease severity of patients was highly correlated with vaccination doses (100% vs. 84.2% vs. 45.4%, *p* = 0.019). The interval between the last vaccination and the viral exposure was significantly different among the three groups (73 vs. 61 vs. 50 days, *p* = 0.047). There was a significant trend toward increasing disease severity with the vaccination interval shortening (*p*_trend_ = 0.003). No significant difference in *C*_t_ values at admission among the three groups was observed (*p* = 0.863).

**Table 1:** Baseline of characteristics of 36 patients with COVID‐19

Characteristics	Total (*N* = 36)	Asymptomatic (*n* = 6)	Mild (*n* = 19)	Moderate (*n* = 11)	*p* value
Age (years)	41.6 ± 11.4	35.7 ± 8.9	41.0 ± 12.2	45.8 ± 10.1	0.205
Gender					
Male	25 (69.4%)	2 (33.3%)	13 (68.4%)	10 (90.9%)	0.035*
Female	11 (30.6%)	4 (66.7%)	6 (31.6%)	1 (9.1%)	
Comorbidity	2	0	1	1	1.000
Days after last vaccination	55 (42−67）	73 (57−103）	61 (42−77）	50 (30−62）	0.047*
Vaccination dose					0.019*
Two doses	27 (75.0%)	6 (100%)	16 (84.2%)	5 (45.4%)	
One dose	9 (25.0%)	0 (0%)	3 (15.8%)	6 (54.6%)	
*C*_t_ value at admission	23.00 ± 8.29	23.11 ± 7.64	24.55 ± 6.68	23 ± 8.29	0.863

Abbreviations: COVID‐19, coronavirus disease 2019; *C*_t_, cycle threshold.

*Note*: **p < 0.01. *p* values comparing three groups are from *F* test, Fisher's exact test, or Kruskal−Wallis *H* test. *p*_trend_ values for trend test are used when appropriate. *p*_trend_ for age was 0.082, *p*_trend_ for vaccination date was 0.003.

* *p *< 0.05.

### 3.2 Numbers of leukocytes, lymphocytes, CRP levels, and IL‐6 concentrations of 36 vaccinated adults infected with SARS‐CoV‐2

As soon as the persons became RT‐PCR positive in the quarantine unit, they were assigned to our hospital regardless of the presence of disease symptoms. They underwent medical and laboratory examinations on Days 1−3, 4−7, 8−14, respectively. Blood samples were taken and the total number of leukocytes, the lymphocyte count, and the presence of CRP, IL‐6 concentrations were determined (Table 2).

**Table 2:** Laboratory findings of 36 patients with COVID‐19 at different time courses

Laboratory findings	Total (*N* = 36)	Asymptomatic (*n* = 6)	Mild (*n* = 19)	Moderate (*n* = 11)	*p* value^a^	*p* value^b^
Leukocytes, ×10^9^/L						
Days 1−3	5.7（4.8−7.0）	5.5（4.7−9.4）	5.9（5.0−6.9）	5.6 (4.8−7.1）	0.977	0.837
Days 4−7	5.5（4.6−6.9）	5.0（4.4−6.0）	5.7（5.0−8.1）	4.8 (3.1−6.1)	0.302	0.630
Days 8−14	6.4（5.5−8.0）	6.8（5.0−7.6）	6.4（5.7−8.2）	6.5 (4.9−7.9)	0.772	0.938
Lymphocytes, ×10^9^/L						
Days 1−3	1.1 ± 0.5	1.1 ± 0.5	1.0 ± 0.4	1.1 ± 0.5	0.932	0.878
Days 4−7	1.5 ± 0.6	1.7 ± 0.5	1.6 ± 0.6	1.3 ± 0.5	0.430	0.430
Days 8−14	1.9 ± 0.6	2.1 ± 0.5	2.0 ± 0.6	1.6 ± 0.5	0.093	0.053
CRP, mg/L						
Days 1−3	7.4 (2.6−17.8）	2.1 (0.5−7.6）	7.9（2.6−19.2）	7.6 (6.9−25.5）	0.268	0.126
Days 4−7	8.8 (2.2−28.6）	2.1 (0.6−6.8）	4.8 (2.2−12.7）	26.6 (14.4−44.5）	0.039*	0.042*
Days 8−14	1.6 (0.8−6.2）	1.4 (0.3−1.7）	1.4（0.8−2.3）	11.2 (1.8−38.1）	0.014*	0.012*
IL‐6, pg/ml						
Days 1−3	6.1 (2.4−0.6）	5.7 (1.7−7.9）	4.4 (2.3−8.2）	9.9 (4.9−17.0）	0.291	0.469
Days 4−7	1.8 (1.3−14.4）	1.2 (1.2−7.4）	1.7 (1.3−2.9）	27.4 (1.8−40.4）	0.010*	0.001*
Days 8−14	1.4 (1.3−2.4）	1.4 (1.2−1.9）	1.4 (1.3−2.1）	1.6 (1.3−11.9）	0.593	0.156

Abbreviations: COVID‐19, coronavirus disease 2019; CRP, C‐reactive protein; IL‐6, interleukin‐6.

* *p *< 0.05, **p < 0.01.

^a^ *p* values comparing three groups are from the *F* test, Fisher's exact test, or Kruskal−Wallis *H* test.

^b^ *p* values for trend test are used.

The median values of leukocytes of all three groups were within the normal range at the three stages. There was a slight decrease on Days 4−7, and this was restored on Days 8−14. No significant differences were found in the leukocyte numbers among the three groups at three stages. A transient lymphopenia was observed at admission, and which disappeared from Days 4−7 on, being less fast for the moderate group. The lymphocyte numbers did not show a significant difference among the three groups (2.1 vs. 2.0 vs. 1.6 × 10^9^/L; *p* = 0.093) on Days 8−14. The CRP levels were highly elevated in the moderate group on Days 4−7 but declined to 11.2 mg/L on Days 8−14. The asymptomatic group and mild group had normal levels. The CRP level on Days 4−7 and 8−14 was significantly different among the three groups (*p* = 0.039 and *p* = 0.014, respectively), and a significant trend of disease severity depending on the elevation of CRP level could be observed (*p*_trend_ = 0.042 and *p*_trend_ = 0.012, respectively). For IL‐6 levels different kinetics could be noticed. The IL‐6 concentrations were within the normal range for the asymptomatic and mild groups. The level of IL‐6 for the moderate group was elevated on Days 1−3, peaked (27.4 pg/ml) on Days 4−7, and then decreased to the normal level on Days 8−14. Significantly different IL‐6 levels were found on Days 4−7 (1.2 vs. 1.7 vs. 27.4 pg/L; *p* = 0.010). Furthermore, with the elevation of the IL‐6 level on Days 4−7, a significant trend toward increasing disease severity was noticed (*p*_trend_ = 0.001).

### 3.3 T‐cell subsets and total antibodies in SARS‐CoV‐2 infected vaccinees

T‐cell subsets including CD3+ CD4^+^ Th and CD3+ CD8^+^ Ts T‐cells, the percentage of Th cells, the percentage of Ts cells, the ratio of Th/Ts, and total antibody level against SARS‐CoV‐2 were determined (Table 3). The number of Th cells in the asymptomatic group (533/μl) and the mild group (736/μl) were both within the normal range. The Th cell numbers in the moderate group were reduced (359/μl). There was no significant difference in the number of Th cells among the three groups (*p* = 0.194). The number of Ts cells was also reduced in the moderate group (520 vs. 340 vs. 281/ul; *p* = 0.072). The percentage of Th cells and Ts cells both were within the normal range. Although a significant difference was found among the three groups for the percentage of Ts cells (32.2 ± 4.0% vs. 22.0 ± 7.2% vs. 29.3 ± 8.0%; *p* = 0.004). The Th/Ts ratio remained within the normal range for all groups but showed a significant difference among the three groups (*p* = 0.028). The moderate group showed the highest antibody titers. The asymptomatic group exhibited modest antibody levels (383, signal‐to‐cutoff ratio [S/CO]), compared to the mild group (703, S/CO) and the moderate group (1792, S/CO). The antibody levels among the three groups were significantly different (*p* < 0.001). There was a significant trend toward increasing disease severity with the increase of antibody titers (*p*_trend_ < 0.001).

**Table 3:** T‐cell counts and total antibody for 36 patients infected with SARS‐CoV‐2

Tests	Total (*N* = 36)	Asymptomatic (*n* = 6)	Mild (*n* = 19)	Moderate (*n* = 11)	*p* value^a^	*p* value^b^
Th cells/ul	627	533	736	359	0.194	0.353
	(437−908）	(511−945）	(490−945）	(260−848）		
Th cells %	39.5 ± 8.2	39 ± 5.0	40.3 ± 9.6	38.3 ± 7.4	0.807	0.866
Ts cells/ul	354 (280−538)	520 (390−686)	340 (280−539)	281 (228−521)	0.702	0.052
Ts cells %	25.9 ± 8.1	32.2 ± 4.0	22.0 ± 7.2	29.3 ± 8.0	0.004**	0.426
Th/Ts ratio	1.6	1.2	2.1	1.3	0.028*	0.720
	(1.1−2.2）	(1.0−1.4）	(1.3−2.6）	(1.0−1.8）		
Ab, S/CO	763	383	703	1792	<0.001**	<0.001**
	(506−748）	(109−488）	(515−928）	(1647−2061）		

Abbreviations: Ab, total antibody; SARS‐CoV‐2, severe acute respiratory syndrome coronavirus‐2; S/CO, signal‐to‐cutoff ratio; Th, helper T‐cells; Ts, suppressor T‐cells.

* *p *< 0.05

** *p *< 0.01.

^a^ *p* values comparing three groups are from the *F* test, Fisher's exact test or Kruskal−Wallis *H* test.

^b^ *p* values for trend test are used.

### 3.4 Ordinal logistic analysis of risk factors for COVID‐19 severity

The results of the univariate ordinal logistic analysis showed that CRP at Days 4−7 and 8−14, IL‐6 at Days 4−7, and total antibody were significantly associated with the infection severity. Furthermore, the multivariate ordinal logistic analysis after adjusting for gender, vaccination date, and vaccination dose indicated that CRP at Days 4−7 (OR = 1.049, 95% CI = 1.005−1.096, *p* = 0.029), CRP at Days 8−14 (OR = 1.161, 95% CI = 1.015−1.327, *p* = 0.029), IL‐6 at Days 4−7 (OR = 1.203, 95% CI = 1.044−1.386, *p* = 0.011), total antibody (OR = 1.006, 95% CI = 1.002−1.010, *p* = 0.007) were risk factors for COVID‐19 severity (Table 4).

**Table 4:** Ordinal logistic analysis of risk factors for COVID‐19 severity

Variables	Crude OR (95% CI)	*p* value	Adjusted OR (95% CI)^a^	*p* value
Leukocytes, ×10^9^/L				
Days 1−3	1.079（0.774−1.504）	0.654	1.098（0.758−1.591）	0.620
Days 4−7	0.886（0.659−1.193）	0.427	1.269（0.874−1.843）	0.211
Days 8−14	0.939（0.635−1.389）	0.752	1.040（0.655−1.651）	0.868
Lymphocytes, ×10^9^/L				
Days 1−3	0.961（0.234−3.948）	0.956	1.461（0.295−7.233）	0.642
Days 4−7	0.471（0.147−1.510）	0.205	0.815（0.188−3.531）	0.785
Days 8−14	0.275（0.082−0.921）	0.036*	0.424（0.088−2.038）	0.284
CRP, mg/L				
Days 1−3	1.036（0.988−1.086）	0.149	1.030（0.980−1.083）	0.242
Days 4−7	1.04（1.003−1.079）	0.035*	1.049（1.005−1.096）	0.029*
Days 8−14	1.128（1.020−1.248）	0.019*	1.161（1.015−1.327）	0.029*
IL‐6, pg/ml				
Days 1−3	0.993（0.955−1.033）	0.740	0.980（0.940−1.021）	0.332
Days 4−7	1.146（1.043−1.259）	0.005*	1.203（1.044−1.386）	0.011*
Days 8−14	1.302（0.959−1.768）	0.090	1.200（0.865−1.664）	0.275
Th cells/ul	0.999（0.997−1.001）	0.210	0.999（0.997−1.001）	0.313
Th cells %	0.988（0.918−1.062）	0.738	0.976（0.898−1.061）	0.569
Ts cells/ul	0.997（0.993−1.000）	0.082	0.997（0.992−1.001）	0.106
Ts cells %	1.008（0.932−1.090）	0.845	0.993（0.910−1.083）	0.868
Th/Ts ratio	0.905（0.496−1.651）	0.746	0.963（0.483−1.921）	0.915
Ab, S/CO	1.006（1.003−1.009）	0.001**	1.006（1.002−1.010）	0.007*

Abbreviations: Ab, total antibody; CI, confidence interval; COVID‐19, coronavirus disease 2019; CRP, C‐reactive protein; IL‐6, interleukin‐6; OR, odds ratio; S/CO, signal‐to‐cutoff ratio; Th, helper T‐cells; Ts, suppressor T‐cells.

* *p *< 0.05

** *p *< 0.01.

^a^ Adjusted for gender, vaccination date, and vaccination dose.

## 4 DISCUSSION

Host responses to SARS‐CoV‐2 infections are heterogeneous, infections with SARS‐CoV‐2 result in asymptomatic presentations to severe outcomes. Several studies show that the Delta SARS‐CoV‐2 variant can be transmitted by asymptomatic individuals.^9^, ^10^, ^11^ The high transmissibility of SARS‐CoV‐2 Delta variant causes a major challenge for public health all over the world.^12^ To cope with this, China encourages mass vaccination for individuals aged 18−60 years. The 36 postvaccination infections with the Delta variant of SARS‐CoV‐2 in this study may reflect on a small scale what happens on a larger scale in the world and help us to understand what are the mechanisms and characteristics of asymptomatic SARS‐CoV‐2 Delta variant breakthrough infections.

In this study, we compared the immunologic features of asymptomatic postvaccination infections by the Delta variant of SARS‐CoV‐2 with similar infections of mild to moderate severity. Previous studies have shown that lymphocyte subset (CD4+ and CD8+ T‐cells) counts reflected the disease severity and were associated with clinical outcome.^13^ Lymphopenia is also mentioned as a predictor of disease severity in COVID‐19.^14^ Our data are consistent with the findings of these studies. Lymphocyte subset counts, and lymphocyte counts were both within the normal range for the asymptomatic group. Several studies have documented the association between COVID‐19 severity and the level of CRP and IL‐6.^15^, ^16^ The data in this study showed a significant increasing trend of disease severity with an elevated level of CRP on Days 4−7, 8−14 and IL‐6 level on Days 4−7. However, the asymptomatic group consistently exhibited a normal level of CRP and IL‐6 at three stages.

A modest level of total antibody in the asymptomatic group was found (383, S/CO). The low titers of antibody may prevent a symptomatic outcome mediated by intensive immune responses.^17^, ^18^ The modest level of antibodies in the asymptomatic group can be explained by the relatively high percentage of Ts cells and the long interval between the last vaccination and infection.

The association between CRP, IL‐6, antibody and SARS‐CoV‐2 infection severity has been reported in several studies.^17^, ^19^, ^20^ Our findings proved that CRP, IL‐6, and total antibody are risk factors for COVID‐19 severity. In the mild and moderate group, 84.21% and 45.45%, respectively of the individuals had two vaccine injections, which demonstrates that partial vaccination is not providing sufficient protection,^21^ and vaccination dose is an independent factor towards COVID‐19 severity. Remarkably, despite that no significant difference in *C*_t_ values at admission among three groups was observed, the *C*_t_ values at the admission of three individuals in the asymptomatic group were less than 25, which suggests that these three asymptomatic individuals may be contagious due to high viral load.^22^

First breakthrough infection studies have focused on functions of the immune responses and breakthrough infections.^23^, ^24^, ^25^ Duarte et al.^26^ described the clinical outcome and immunological profile of vaccine breakthrough infection cases, fully vaccinated, and showed that breakthrough cases had a good T‐cell response. Another report evaluated breakthrough infections and humoral responses.^27^ The antibody responses were significantly higher in symptomatic individuals with breakthrough infections but were not elevated for asymptomatic breakthrough infections. Our study is consistent with these reports. The asymptomatic breakthrough infection group in our study has normal cellular immunity together with modest humoral immunity. This might imply that cellular immunity may play a more important role in protective immunity to SARS‐CoV‐2 infections than humoral immunity.^28^

This study has some limitations. First, this is a retrospective, single‐center study, and with a small number of participants. Second, the baseline for Th and Ts cell counts, and the total antibody levels were not available. The impacts of vaccination or previous infections on Th cells counts, Ts cells counts, and total antibody levels can not be ruled out.

## 5 CONCLUSION

Data from this outbreak suggest that asymptomatic infections are prone to occur in fully‐vaccinated individuals. The immunophenotype of the infections in the vaccinated asymptomatic patients is less inflammatory, accompanied by a modest antibody response. This information will help to understand the immune status of asymptomatic postvaccination infections.

## AUTHOR CONTRIBUTIONS

Rui Duan and Pei Wang wrote the manuscript. Qiang Mao was the chief investigator and was responsible for the data analysis. Rui Duan, Qiang Mao, Xu Ding, Qiwu Qiu, and Pei Wang discussed the data. All authors contributed to the writing of the final manuscript. All authors have approved the final version of the manuscript for publication.

## CONFLICT OF INTEREST

The author declare no conflict of interest.

## ETHICAL STATEMENT

This study was approved by the Ethics Committee from The First People's Hospital of Jingmen (ID: 202102090). Written informed consent was waived by the Ethics Commission of the designated hospital for emerging infectious disease.

## Data Availability

The data that support the findings of this study are available upon reasonable request from the corresponding author Dr. Pei Wang.
